# Development of an Instrument to Assess Spirituality: Reliability and Validation of the Attitudes Related to Spirituality Scale (ARES)

**DOI:** 10.3389/fpsyg.2021.764132

**Published:** 2021-11-04

**Authors:** Camilla Casaletti Braghetta, Clarice Gorenstein, Yuan Pang Wang, Camila Bertini Martins, Frederico Camelo Leão, Mario Fernando Prieto Peres, Giancarlo Lucchetti, Homero Vallada

**Affiliations:** ^1^Departamento de Psiquiatria, Faculdade de Medicina (FMUSP), Instituto de Psiquiatria, Universidade de São Paulo, Programa de Saúde, Espiritualidade e Religiosidade, São Paulo, Brazil; ^2^Laboratório de Psicopatologia e Terapêutica Psiquiátrica (LIM-23), Departamento de Psiquiatria, Faculdade de Medicina (FMUSP), Instituto de Psiquiatria, Universidade de São Paulo, São Paulo, Brazil; ^3^Departamento de Farmacologia, Instituto de Ciências Biomédicas, Universidade de São Paulo, São Paulo, Brazil; ^4^Departamento de Medicina Preventiva, Escola Paulista de Medicina, Universidade Federal de São Paulo, São Paulo, Brazil; ^5^School of Medicine, Federal University of Juiz de Fora, Minas Gerais, Brazil

**Keywords:** spirituality (MeSH), scale, factorial analisys, instrument, measure, psychometrics

## Abstract

**Background:** Several instruments that measure spirituality present overlaps with positive emotions, impacting the interpretation of their findings. In order to minimize these problems, we aimed to develop, assess the reliability and validate a new scale to evaluate spirituality.

**Methods:** The instrument was designed using a theoretical framework minimizing tautological issues (i.e., Koenig’s framework), a qualitative study investigating the definitions of spirituality, the development of the first version of instrument by experts’ meetings and a qualitative cognitive debriefing. Then, the instrument was examined for its content validity by a multidisciplinary group of judges and was pilot-tested in two different groups – less religious (medical students – *n* = 85) and more religious (practicing religious members – *n* = 85). Finally, psychometric properties and validity were assessed.

**Results:** The developed Attitudes Related to Spirituality Scale (ARES) is a self-report 11-item instrument using five-level Likert items. ARES presented appropriate psychometric properties revealing excellent internal consistency (alpha = 0.98) and temporal stability (ICC = 0.98). Likewise, ARES was strongly correlated with other validated R/S instruments (i.e., Duke Religion Index and Brief Multidimensional Measure of Religiousness/Spirituality) and was able to discriminate higher and lower religious groups. In the exploratory factor analysis, a unidimensional structure of the scale was described. Fit indices for the scale demonstrated good fit in the unidimensional model.

**Conclusion:** The ARES is a reliable, valid and stable one-dimension instrument that is appropriate for use in the Portuguese-speaking population.

**Descriptors:** Spirituality; Scale; Factorial Analysis; Instrument; Measure; Psychometrics.

## Introduction

A growing number of publications have examined spirituality and religiosity (S/R) and their relationship to health, generally showing favorable effects of spiritual beliefs on both physical and mental health (Sawartzky et al., 2005; Moreira-Almeida et al., 2014; Bai and Lazenby, 2015). Likewise, spiritual and religious practices have an impact on individuals’ lifestyles showing an effect on moral and ethical values (Gonçalves et al., 2015).

Spirituality is a complex concept and, by involving subjective experiences, many individuals have their own definition of this term. Even those who share the same cultural and social experiences may have different ways of understanding and expressing their spirituality. Historically, spirituality was strongly linked to religion (Koenig, 2008). The contemporary view of spirituality and recent studies have shown the use of the term spirituality detached from religion and religiosity, and the emergence of “spiritual but not religious” individuals (Larson et al., 1998). Spirituality and religiosity are overlapping constructs, but most researchers agree that there is a difference between them. In a study on concepts about these constructs, an in-depth content analysis was conducted by the authors about definitions of spirituality, religiousness, faith, and the sacred. It was observed that spirituality is more specifically related to the search for or to the relationship with the sacred, while religiousness is considered a ritual, institutional or codified spirituality that is culturally sanctioned (Harris et al., 2018).

There are several definitions of spirituality being used in the literature without a consensus. Some authors adopted a narrower view, in which spirituality is necessarily linked to the sacred or transcendence such as noted in the definition provided by Harold Koenig (“a personal search for understanding were related to larger existential issues, i.e., the meaning of life, death; and its relations with the sacred and/or transcendent, without implying the formation of religious communities”) (p. 18) (Koenig et al., 2012). In contrast, other authors adopted a broader view, which includes other aspects such as nature, arts, and family in the concept of spirituality. According to Puchalski and Romer (2000), spirituality is what “allows a person to experience a transcendent meaning in life, expressed as a connection with God, but including the relationship with nature, arts, music, family or community, or any beliefs and values give a person a sense of meaning and purpose in life” (p. 129). This discussion is seldom solved and is the target of several articles in recent years (Hill et al., 2000; MacDonald et al., 2015). Pargament and Mahone (2009) underscores that, although it is clear that spirituality differs from religion as an individual expression, adopting a broader view can have the problem of losing the “sacred core” of this conceptual field.

The present study was based on Koenig’s definition of spirituality, as described above, and on the challenges of quantifying spirituality in clinical research. Koenig’s definition tends to be more delimited concerning the central core of the concept of spirituality, which involves aspects related to the “sacred” and the “transcendent”. It is important to point out that spirituality has, in part, an association with religious involvement, as spirituality can be considered as a way of life, which influences an individual’s worldview, decisions, and behaviors (Koenig et al., 2012). However, Koenig’s definition also allows the separation of those “spiritual but not religious” individuals which are those with spiritual beliefs, but not necessarily related to the involvement with religious communities (Koenig, 2008). Based on this theoretical model, it is important to use a definition that allows for a better examination of the relationship between spirituality and health in studies.

In summary, spirituality is a complex subjective concept, and it is necessary to quantify it for use in research, however, this has been a major challenge. Instruments that include broad definitions of spirituality in their theoretical basis (embracing aspects such as the feelings of tranquility, harmony, optimism, forgiveness, peace, and general well-being) can be considered problematic since they can overlap with psychological well-being measures and positive characteristics of mental health (Moreira-Almeida et al., 2006). This controversy over measurement implies a tautological question because the inclusion of indicators of psychological well-being in instruments to assess spirituality results in a positive correlation between spirituality and well-being. Another problem is the experience of secular individuals, who may also experience a sense of peace and harmony without necessarily being involved with the issue of spirituality. Extremely broad definitions led to the impossibility of differentiation, since practically all individuals can be considered spiritual, and as such, relations with mental health and behaviors cannot be studied (Larson et al., 1998).

The problem of tautology can be verified in different scales that are used worldwide. Tautology refers to the use of spirituality scales that contain “contaminated” items (i.e., items that assess positive experiences or psychological well-being of individuals). In other words, tautological instruments can be considered of limited value for research because, by definition, they can be expected to be predictably correlated with items that elicit information about psychological well-being. The Functional Assessment of Cancer Therapy (FACIT-SP; Peterman et al., 2002), for example, is a scale usually used in the oncological context and presents items such as “I feel peaceful”, “I am able to reach down deep in myself for comfort” and “I feel a sense of harmony within myself”. Another frequently used scale, the Daily Spiritual Experiences Scale (DES; Underwood and Teresi, 2002) includes statements such as: “I feel a deep inner peace or harmony”, “I feel a selfless caring for others” and “I accept others even when they do things that I think are wrong”. The items mentioned above for these scales validated in Brazil could be characterized as a tautological issue because what they intend to evaluate is much more related to concepts of psychological well-being than spiritual issues. This is because they have used very broad concepts of spirituality in their definitions, that encompass other positive experiences. This is a problem since patients with severe depression or anxiety, for instance, will not consider themselves peaceful and, for this reason, will not be considered spiritual in such scales, even if they have spiritual beliefs. When we do not consider the tautological concept, we indirectly assume that only spiritual persons experience peace, harmony, and care deeply about others, and for this reason, the spirituality assessment is overlapping with its outcome (Moreira-Almeida et al., 2006; Koenig, 2008).

Koenig et al. (2012) points to possibilities for developing non-tautological measures of spirituality. First, the instrument should not include items that clearly tap positive psychological aspects or are related to mental health (feelings of peace, tranquility, harmony, and comfort). Second, spirituality should be measured using questions about beliefs, practices, attitudes, degree of commitment, and level of motivation. According to the author, this will allow for a better delineation between religion, spirituality, and health without confusion.

In this context, new instruments that are based on non-tautological frameworks of spirituality are welcome in the literature. Even though other instruments are assessing this construct, most of them were developed in high-income countries with Anglo-Saxon backgrounds (Monod et al., 2011; Sessanna et al., 2011; de Jager Meezenbroek et al., 2012), which are developed under different cultural visions and assumptions. Therefore, culturally adapting available international instruments could result in biases of interpretation for middle to low-income populations.

Unfortunately, in our search of the literature, instruments designed to be applied in developing countries and with different cultural backgrounds are yet scarce. Furthermore, a systematic review carried out in the Brazilian context found twenty instruments to assess spirituality and religiosity for health research in Brazil and Portugal. It was observed that most of the instruments mentioned in that review, according to the authors’ assessment, do not present all the psychometric properties (Lucchetti et al., 2013b). In addition, none of the instruments questioned the tautological issue of the scales of spirituality, representing an important gap.

Advances in new instruments in this area could contribute to a closer examination of the relationship between physical health, mental health, and spirituality (Larson et al., 1998). To bridge this gap, the present study aimed to develop, assess the reliability of and validate a new scale to evaluate spirituality in the Brazilian context, named the ARES – Attitudes Related to Spirituality Scale.

## Materials and Methods

To create a new instrument to assess spirituality in Brazil, two different phases were followed. The first phase was the instrument’s development, and the final phase consisted of validity and reliability studies of the developed instrument.

### Phase 1: Development of the Instrument

The instrument was developed in four stages:

(a) Theoretical framework: for the present study, aiming to minimize the tautological problems of previous instruments as discussed previously, we adopted the concept of spirituality proposed by Koenig (Koenig et al., 2012), which is based on a definition of the term spirituality as a more transcendental and sacred dimension and allows for differentiation of spiritual experiences as opposed to religious practices, including those who consider themselves “spiritual”, but not as “religious” individuals (Koenig, 2008). The theoretical model was decisive for the construction of the scale, as it minimized the overlapping with other constructs such as positive characteristics of mental health and psychological well-being.

(b) Qualitative study for item generation and construction: To develop the items of ARES, a qualitative study including a convenience sample of 60 individuals was carried out, following our theoretical choice of reference (i.e., non-tautological theory). The objective of this preliminary study was to assess how this population understood spirituality and to contribute to the development of the items using the question “How do you define spirituality?”. Tautological answers (e.g., answers overlapping with well-being and mental health) were excluded since they did not support our theoretical framework (see above). In this study, the sample consisted of graduate and undergraduate students and employees from different professional categories and different cultural backgrounds from a public university who were approached personally, invited at that time, and voluntarily agreed to participate. The inclusion criteria were individuals over 18 years old who agreed to participate and signed the informed consent form and all the invited individuals agreed to participate. Of this sample, 52% of the participants were female, with a mean age of 39 years (SD = 16 years). In terms of schooling, 35% of the participants had completed higher education. The interviews were conducted face-to-face by the first author in a single meeting taking approximately 20 min. Answers to the target question were analysed according to Bardin’s content analysis method (Bardin, 2009). It uses thematic categories, trying to detect elements and then dividing them into categories, identifying what they have in common. The content analysis technique consists of three stages: pre-analysis, exploration of the material and treatment of the results/interpretation. In the first stage, also known as the organization phase, we used a reading of the material and the initial survey of hypotheses that could guide the final interpretation, based on existing concepts of spirituality and discussed in the literature. In the second stage, the data was encoded from the registration units, creating categories. The categories identified based on the literature on spirituality were spirituality as transcendence, spirituality as religious beliefs and spirituality as positive emotions and feelings. In the third stage, the elements were classified according to their similarities, allowing their grouping into the discriminated categories. In this last phase, which is also known as the data interpretation phase, it was necessary to return to the theoretical framework used in this work, seeking to support the analyses, giving meaning to the interpretation.

(c) Development of the instrument: based on the material collected in the steps described above, a committee of experts was invited to carry out the operationalization of the items of the first version of the scale. The committee was composed of five professionals, who were invited based on their experience in spirituality research (an occupational therapist and two psychiatrists) or in the field of psychometrics (a psychiatrist and a pharmacologist). They created a set of items with the potential to assess spirituality. These were generated from concepts discussed in the existing literature (theoretical model adopted for this study) and based on statements obtained through the qualitative study of definitions of spirituality. With the above considerations, the first version of the scale was constructed, aiming to be brief, non-tautological and adhering to the conceptual framework. The items were revised; the redundant or biased items were eliminated. More details concerning the statements and items are provided below and in the Supplementary Material Additional file 1.

(d) Test for understanding of the items (qualitative cognitive debriefing): the objective of this stage was to test the understanding and to make a semantic analysis of the proposed items. Face-to-face interviews were conducted with 30 participants (53.3% women) from a non-probabilistic sample formed by a cleaning and maintenance team from a university hospital. The interviewees’ ages ranged from 18 to 58 years (*M* = 35 years and SD = 13 years). Of the total sample, 23% had nine years of schooling and 77% had 12 years of schooling. The participants were presented one item at a time; they then discussed any doubts and were asked for suggestions on the formulation of the items.

**FIGURE 1:**
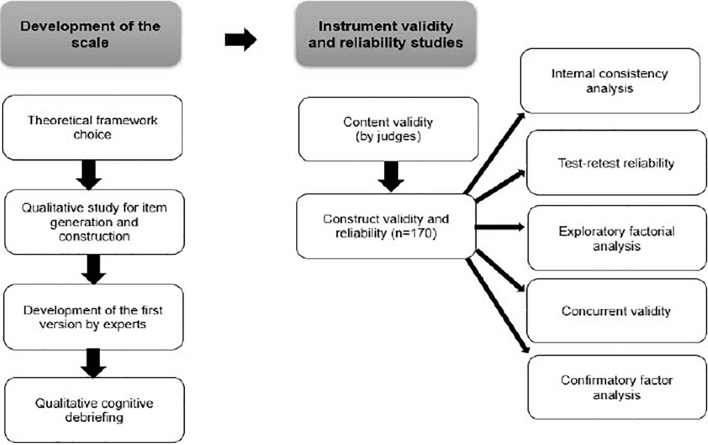
Diagram describing the phases of the study and the ARES development.

Figure 1 shows all the phases of the study and the ARES development.

### Phase 2: Instrument Validity and Reliability Studies

(a) Content validity: Six judges were invited, consisting of three psychologists and three physicians, each of whom had expertise in the areas of spirituality and psychometrics. This panel of judges was composed of distinguished professionals from the committee that was invited to develop the items. The study proposal and the instrument in its full version were presented to these judges through an online form. These items were questioned, item-by-item, on the following aspects: (a) whether the item in question assessed what it intended to measure (**concordance index** – CI) and (b) whether the item was relevant or representative within the subject (**content validity index** – CVI; Alexandre and Coluci, 2011). These procedures measured the proportion of the judges who were in agreement regarding the content of the instrument and its items. A minimum agreement of 80% on each index indicated the validity of the scale. Where relevant, the judges suggested changes in the wording of the items of the instrument. The final version of the scale was translated into English by two native translators of a translation company (a British and an American translator) and is available below in the Supplementary Material Additional file 2.

(b) For the psychometric analysis of construct validity and reliability, two contrasting groups were recruited from non-clinical settings: a higher religious group composed of 85 religious assistants (volunteers who were responsible for spiritual and religious assistance) from a religious center and a lower religious group composed of 85 students of the School of Medicine of the University of São Paulo, Brazil (*n* = 170). Medical students were chosen because they have lower levels of religiousness as compared to the general population in Brazil (Lucchetti et al., 2013a). Moreover, the choice of students was considered because it is a more homogeneous group, coming from the same institution, as opposed to trained professionals, who could have different backgrounds and coming from different services. Construct validity’s objective is to assess whether the instrument’s items constitute a legitimate representation of the construct. Thus, this analysis aims to assess whether the instrument’s items are related, justifying their grouping to represent the dimensions of the construct (American Psychological Research Association, 1954). The sample size calculation was based on previous guidelines assessing sample sizes for validation studies, which consider the number of at least ten respondents for each item of the questionnaire (Hair et al., 2005; Anthoine et al., 2014). Using this guideline, the minimum sample size for the 11-item ARES scale was 110 participants. In the test-retest reliability analysis, the only ones who participated were the students (*n* = 67) who had answered the scale again after 15 days, since it was understood that the scale scores would certainly be stable in the high religious sample. Concurrent validity was assessed correlating ARES with other Brazilian validated S/R instruments [Duke Religion Index (Lucchetti et al., 2012) and the Brief Multidimensional Measure of Religiousness/Spirituality (Curcio et al., 2015)].

The Research Ethics Committee of the Medical School of the University of São Paulo approved the research protocol (no. 214/15) for the fieldwork and data collection. The participants were informed that the data would be treated with strict confidentiality and that no personal information would be disclosed. Participation was voluntary, and there was no form of compensation.

### Statistical Analysis

The CVI used a Likert scale with a score of 1 to 4 to evaluate the relevance/representativeness of the answers, ranging from 1 = non-representative to 4 = relevant or representative item. Only the items marked by the judges with values of 3 and 4 were considered evidence of content representativeness (Grant and Davis, 1997). For all the following analyses, a sample with 170 participants was considered. For the construct validity, the possibility of conducting a factor analysis was verified by the Kaiser-Meyer-Olkin (KMO) criterion and Bartlett’s sphericity test. The method of estimation of the factorial model that was used was Principal Axis Factoring (PAF). PAF is an exploratory approach and probably the most widely used method for factor analysis (Warner, 2012). It was used to find a structure of interrelations between the observed variables, determining the number and nature of the factors that best represent these variables. The criteria for determining the number of factors to be extracted was Kaiser’s rule (eigenvalue > 1). Factor loadings above 0.30 were used as the criteria for retaining an item in each factor (Warner, 2012). Cronbach’s alpha coefficient (Cronbach, 1951; Brown, 2002) was used to evaluate the internal consistency of the questionnaire. Kendall’s coefficient of agreement and the intraclass correlation coefficient (ICC) were used to evaluate the test-retest reliability (Kendall et al., 1939; McGraw and Wong, 1996). Concurrent validity was assessed correlating ARES with other Brazilian validated R/S instruments using Spearman correlation coefficients (rho). In the discriminant validity, spirituality scores were compared for both groups using Mann–Whitney tests. Likewise, scores were classified into low and high spirituality/religiousness using the median values of the whole sample for each scale. Discriminant validity between known groups is a form of validity that aims to identify differences between groups in which these differences are theoretically expected to be found, using the hypothesis that groups of individuals perceived as different in relation to the construct to be measured, produce different values when the instrument is applied. This type of validity aims to assess the presence of differences in the measurements obtained between the groups, not whether the measure really measures the intended construct (Echevarría-Guanilo et al., 2018). Confirmatory factor analysis (CFA) with estimation by the robust weighted least squares (WLSMV) was used to provide evidence for construct validity. The Comparative Fit Index (CFI), the Tucker-Lewis Index (TLI), and Root-Mean-Square Error Approximation (RMSEA) were used to evaluate goodness-of-fit based on the following cutoff criteria: RMSEA estimate around or less than 0.08, and CFI and TLI greater than 0.90. Factor loading shows the variance explained by the variable on each factor of the model. We considered that factor loadings greater than 0.7 provide evidence that the factor extracts sufficient variance from that variable (Brown, 2006). Logistic regression models were carried out (high versus low scores on ARES) adjusting for gender (female/male), group (Religious/Medical students), age, marital status (married/not married), education (complete higher education/other) and income (up to 10 salaries/10 or more salaries). The analyses were performed by the Mplus, version 8.0. The data were analysed using the Statistical Package for Social Sciences (SPSS) 16.0 for Windows for all procedures, except for the Kendall’s coefficient of agreement that was assessed using the program R, version 3.3.3. The level of significance considered was 5% (Hope, 1968).

## Results

### Development of the Instrument

In the qualitative study for item generation and construction, according to the answers obtained from the participants, the material content analysis provided in the Supplementary Material Additional file 1, identified three categories of responses:

– First category (Spirituality as transcendence): respondents (*n* = 27) reported that spirituality is linked to the conception of transcendence, that is, there is something or someone beyond daily physical existence that is the source of support. The following was among the representative reports: “Spirituality is man’s belief in something greater than the material reality that surrounds him’. “It is linked to faith, but not necessarily linked to religion”. “It’s something beyond the material world, where the spirit remains alive, to obtain personal evolution”. “Connection with something or higher being, which brings meaning to our existence” (see Supplementary Material Additional file 1).

– Second category (Spirituality as religious beliefs): respondents (*n* = 15) reported that their spiritual experience or their understanding of spirituality comes from beliefs or from a religious tradition. The following was among the most relevant definitions: “It is something that is connected to the contact with God, to the religiosity, to the fact that the person comes into contact with God through prayer”. “It’s a balance and understanding of the relationship between religiosity and me”. “It’s what everyone feels about religion” (see Supplementary Material Additional file 1).

– Third category (Spirituality as positive emotions and feelings): respondents (*n* = 18) cited concepts similar to the definition of secularization, that is, they did not relate the concept of spirituality to religion or to a transcendent being but rather linked spirituality to positive emotions and feelings, doing good to others, greater awareness and ethics. The following was among the reports: “It is to have a positive or negative affect, it is linked to emotions, to the spirit itself”. “Believe or have faith in yourself”. “It is the thought of each person, to be aware of what we have to learn in this world”. “Feeling good about yourself” (see Supplementary Material Additional file 1).

The definitions of spirituality proposed by the participants in the first and second categories (70% of the sample) were related to the chosen theoretical frameworks, showing that the population understood spirituality elements such as the belief in a reality beyond the material (transcendent), through connection with a superior force and, in an independent and sometimes overlapping way, the question of religion. The definitions proposed by the third category did not match the theoretical model chosen for the development of the scale, so they could result in items with tautological questions. Then, it was decided not to consider these statements for the development of the instrument.

From the answers of the participants in the qualitative study, 48 statements that fit the theoretical framework proposed for the study were selected. These statements generated 12 items (see Supplementary Material Additional file 1). Some contents were repeated in many answers of the participants and, because of this, the number of items was limited. No additional information was provided that could generate other items.

In the interviews to get an understanding of items, 30 individuals were approached. After analysing item by item, the participants noted the ones that generated the most doubt. The “Instructions” section was highlighted by the participants as the most difficult to understand, as well as the following items on the scale: “I have spiritual beliefs and values,” “I have had unusual experiences that may have been spiritual experiences”, “Spirituality leads me to have a positive connection with people” and “My life has a spiritual purpose”. The rest of the items reached an appropriate understanding of at least 80% of the participants.

The results of the study were presented to the committee of experts, who analysed the suggestions of the participants and made modifications in the writing of the items that presented greater difficulty of comprehension. Based on the appreciation and amendments proposed by the committee of experts, a second version of the instrument was produced.

### Psychometric Analysis

For the validity of the content, the full scale consisting of 12 items was presented to the judges. According to the judges’ answers, items 5, 8, and 9 of the scale (“Spirituality encourages me to help others”, “I have had experiences that I could not explain, which may have been spiritual experiences” and “I believe in life after death”, respectively) did not reach the minimum of 80% in the CVI, as delineated in the methods (Alexandre and Coluci, 2011). Item 8, for not having reached the minimum of 80% in both the CI and the CVI, was eliminated from the instrument. It was decided that items 5 and 9 would be kept since changes were made to their wording that resulted in greater relevance, a decision that reached agreement among the judges regarding the adequacy of the construct that was intended to be evaluated. However, changes were made to the wording of these according to the judges’ suggestions. After the evaluation and improvement proposed by the judges, the instrument was discussed and approved by the committee of experts, and the final version with 11 items was submitted to the next psychometric analysis.

For the following validation and reliability analyses, students and religious assistants were approached (*N* = 170). ARES is a 11-item-Likert scale whose values vary from 1 (Totally Disagree) to 5 (Totally Agree) for each item. The possible scores range from 11 to 55. Among the respondents, there were a total of five missing observations, which were replaced by the category “Neither agree nor disagree”.

Regarding the characteristics of the sample, the ages of the religious group varied between 29 and 82 years, with an average of approximately 60 years (SD = 1.3 years). In the group of students, the ages ranged from 20 to 36 years, with a mean of 23 years (SD = 0.2). Table 1 shows the sociodemographic characteristics of the samples. All variables were significantly different (*p* < 0.05) between groups.

**TABLE 1:** Sociodemographic characteristics of the samples.

Variables	Total (*n* = 170)		Re (*n* = 85)		Med (*n* = 85)	
	Mean	SD	Mean	SD	Mean	SD
Age	41.6	20.1	59.8	11.6	23.4	2.3
	n	%	n	%	n	%
Gender*						
Male	81	47.6	32	37.6	49	67.6
Female	89	52.4	53	62.4	36	42.4
**Occupation §**						
Employee	45	26.5	43	50.6	2	2.3
Unemployed	1	0.6	1	1.2	0	0.0
Work without remuneration	3	1.8	2	2.3	1	1.2
Retired	39	22.9	39	45.9	0	0.0
Only studying	82	48.2	0	0.0	82	96.5
**Marital Status §**						
Not married	112	65.9	29	34.1	83	97.6
Married	43	25.3	41	48.2	2	2.4
Divorced	12	7.0	12	14.1	0	0.0
Widower	3	1.8	3	3.6	0	0.0
**Schooling §**						
Less than 4 years	3	1.8	3	3.5	0	0.0
9 years	3	1.8	3	3.5	0	0.0
Less than 12 years	4	2.4	4	4.7	0	0.0
12 years	17	10.0	16	18.8	1	1.2
Incomplete university education	82	48.0	3	3.5	79	92.9
Complete university education	61	36.0	56	66.0	5	5.9
**Income + §**						
Less than 1 minimum wage	1	0.6	0	0.0	1	1.2
1 to 2 minimum wages	8	4.8	6	7.1	2	2.4
2 to 3 minimum wages	17	10.1	12	14.1	5	6.0
3 to 5 minimum wages	29	17.3	19	22.3	10	12.0
5 to 10 minimum wages	37	22.0	23	27.1	14	17.0
10 to 20 minimum wages	44	26.2	19	22.3	25	30.1
More than 20 minimum wages	32	19.0	6	7.1	26	31.3
Religious Affiliation*						
No	39	23.0	0	0.0	39	46.0
Yes	131	77.0	85	100.0	46	54.0
**Which § ±**						
Catholic	27	20.6	0	0.0	27	58.7
Evangelical	5	3.8	0	0.0	5	10.9
Spiritism	92	70.2	85	100.0	7	15.2
Afro-brazilian religions	1	0.8	0	0.0	1	2.2
Others	6	4.6	0	0.0	6	13.0

*n, absolute frequency; %, percentage; SD, standard deviation; Re, religious assistants; Med, medicine students.*

*+ Two participants did not report their income, both medical students.*

*± Total of 131 participants who answered Yes in religious affiliation.*

As a result of the internal consistency analysis, the Cronbach’s alpha coefficient was found to be 0.98, indicating the level of homogeneity of the scale items (see Supplementary Material Additional file 3).

For the test-retest reliability analysis, the scale was again applied to a group of 67 medical students 15 days after the initial test. The Intraclass Correlation Coefficient (ICC) was obtained considering the total scores, resulting in an ICC of 0.98 (95% CI = 0.97 – 0.99). For the results obtained from the test-retest agreement of each item, it was verified that the concordance of all items was relatively high and significant (*p* < 0.05) (see Supplementary Material Additional file 4).

The factorial structure of the questionnaire was verified through an exploratory factorial analysis (Table 2), considering the responses of the first application of the 11 items in all the individuals. According to the set of criteria considered in this analysis, a single factor was extracted, explaining 86.69% of the total variability of the data and showing its one-dimensional structure. A high correlation was observed between the items of the scale.

**TABLE 2:** Exploratory factor analysis for the ARES (*n* = 170).

Domain assessed by each item		Factor

Portuguese original version	English translated version*	Factorial load
*Item 1 – Eu acredito em algo sagrado, transcendente (Deus, uma força superior).*	Item 1- I believe in something sacred or transcendent (God, a higher force).	0.91
*Item 2 – Meditação, oração, leituras e/ou contemplação são práticas que utilizo (ao menos uma delas) para me conectar com uma força espiritual além de mim.*	Item 2 – Meditation, prayer, readings and/or contemplation are practices that I use (at least one of them) to connect with a spiritual force beyond myself.	0.92
*Item 3 – Já presenciei fatos/situações que me levaram a acreditar que existe algo além do mundo material.*	Item 3 – I have witnessed facts/situations that have led me to believe that there is something beyond the material world.	0.88
*Item 4 – Minha fé ou crenças espirituais me dão apoio no dia-a-dia.*	Item 4 – My faith or spiritual beliefs sustain me on a daily basis.	0.96
*Item 5 – Minha espiritualidade me ajuda a ter um relacionamento melhor com os outros.*	Item 5 – My spirituality helps me have a better relationship with others.	0.95
*Item 6 – Minha espiritualidade influencia minha saúde física e mental.*	Item 6 – My spirituality influences my physical and mental health.	0.94
*Item 7 - Minha espiritualidade me incentiva a ajudar outras pessoas.*	Item 7 - My spirituality encourages me to help others.	0.94
*Item 8 – Eu acredito em uma continuidade após a morte.*	Item 8 – I believe in continuity after death.	0.91
*Item 9 – Minhas crenças e valores espirituais direcionam minhas ações no dia-a-dia.*	Item 9 – My spiritual beliefs and values guide my day-to-day actions.	0.91
*Item 10 – Minha fé ou crenças espirituais dão sentido à minha vida.*	Item 10 – My faith or spiritual beliefs give meaning to my life.	0.94
*Item 11 – Práticas espirituais (por exemplo: fazer orações, ou jejum, ou meditação ou outras) ajudam a manter ou melhorar a minha saúde física ou mental.*	Item 11 – Spiritual practices (e.g., praying, fasting, meditation or other) help maintain or improve my physical or mental health.	0.85
Eigenvalue		9.53
% variance		86.6
KMO		0.95
Bartlett’s Test		*p* < 0.001

*The American Journal of Experts – AJE provided the English translated version of the items.

Concerning the concurrent validity, ARES score was significantly correlated with other validated R/S scales, presenting high correlation with BMMRS-P (rho = 0.88) and the Duke Religion Index (rho = 0.90) (Table 3).

**TABLE 3:** Correlation between attitudes related to spirituality scale, duke religion index and brief multidimensional measure of religiousness/spirituality.

	ARES	BMMRS-P	DUREL
ARES	1.00	0.88 (*p* < 0.001)	0.90 (*p* < 0.001)
BMMRS-P		1.00	0.89 (*p* < 0.001)
DUREL			1.00

Finally, ARES was able to significantly differentiate the high religious and the low religious groups, as observed with Duke Religion Index and Brief Multidimensional Measure of Religiousness/Spirituality as well (Table 4). The results were maintained even after adjusting for gender, age, marital status, education level, and income (see Supplementary Material Additional file 5).

**TABLE 4:** Summary measures for scale scores brief multidimensional measure of religiousness/spirituality, duke religion index and attitudes related to spirituality scale.

Score	Group	N	Q1	Median	Q3	Mean	Standard Error	*p*-Value
BMMRS-P*	Re	82	71.0	75.0	78.0	74.4	0.6	<0.001
	Med	79	32.0	48.0	61.0	47.3	1.7	
	**Total**	**161**	**48.0**	**68.0**	**75.0**	**61.1**	**1.4**	
DUREL*	Re	83	14.0	15.0	15.0	14.5	0.1	<0.001
	Med	85	3.0	7.0	11.0	7.2	0.5	
	**Total**	**168**	**6.8**	**13.0**	**15.0**	**10.8**	**0.4**	
ARES*	Re	85	54.0	55.0	55.0	54.6	0.1	<0.001
	Med	85	18.0	40.0	47.0	34.5	1.7	
	**Total**	**170**	**40.0**	**53.5**	**55.0**	**44.5**	**1.1**	

N, sample size; Q1, first quartile; Q3, third quartile; Re, religious assistants; Med, medicine students. *Mann–Whitney test.

**FIGURE 2:**
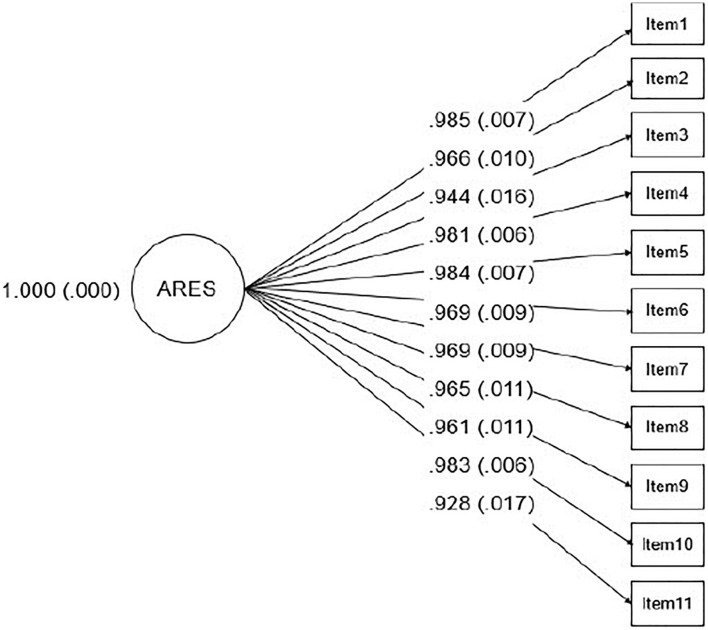
Diagram of the latent model, representing one factor solution for ARES, with standardized factor loadings and their standard errors in parentheses.

Fit indices for the scale demonstrated good fit in the unidimensional model, with X^2^ = 67.008 and *p*-value 0.0143, RMSEA estimate = 0.055, CFI = 1.000, and TLI = 0.999. All the factors loading showed greater values than 0.9 (Figure 2).

## Discussion

The present study developed and validated a new measure to assess spirituality in the Brazilian context, the ARES (see the complete version of the scale in Portuguese and the proposed English version in the Supplementary Material Additional file 2). This instrument had conceptual and temporal stability, was able to discriminate the religiousness and spirituality of the participants and correlated well with other measures. To our knowledge, ARES is the first instrument focusing on non-tautological aspects to be developed in the Portuguese language and one of the few instruments originally developed from participants from middle and low-income countries.

Since ARES does not include tautological aspects, it could be an important tool for the advancement of this field of research, since most of the previous instruments to assess spirituality include items that overlap with aspects of positive mental health. This confusion and overlap were observed in our qualitative study where part of the sample considered spirituality as a synonym of positive emotion. According to the theoretical framework used in ARES, this relationship could impair the correct interpretation of the findings of the spirituality studies and have been extensively discussed by authors (Moreira-Almeida et al., 2006; Koenig, 2008).

Regarding the psychometric analysis of the ARES, it is essential to verify the psychometric analysis of an instrument so that one can choose a valid and reliable measure for use in both clinical and research contexts (Lucchetti et al., 2013b). In this study, Cronbach’s alpha coefficient was high, indicating an appropriate internal consistency of the instrument. Although using different instruments, our results are comparable to other validated spirituality assessment tools for the Portuguese language, which presented satisfactory to excellent internal consistencies: Daily Spiritual Experiences Scale (alpha = 0.91) (Kimura et al., 2015), Brief Multidimensional Measure of Religiousness/Spirituality – BMMRS-P (alpha = 0.88) (Lucchetti et al., 2012), FACIT-Sp (alpha = 0.89) (Lucchetti et al., 2015) and Spiritual Well-being Scale (alpha = 0.92) (Marques et al., 2009). Likewise, the test-retest analysis proved to be high and comparable to previous published instruments (Kimura et al., 2015; Lucchetti et al., 2015). In the analysis of the main components, it was observed that ARES has a one-dimensional structure, allowing researchers to infer that the greater the sum of the scores of the items is, the greater the individual’s spirituality.

Regarding the methodological limitations, first it is important to note that, for the qualitative study of the definitions of spirituality, a convenience sample was selected, which may have resulted in a selection bias. For a future study, a representative Brazilian sample should be recruited to better understand the spirituality profile of this population. Second, in our study, the content analysis was performed by only one researcher. Although this could be considered a potential limitation, qualitative researchers underscore that content analysis can be performed by only one researcher as well (Harding and Whitehead, 2013). However, we understand that the cross-validation of the analysis would make the study more rigorous. Third, we obtained a smaller number of items in the participants’ responses, as we only used the responses that corroborated the theoretical model adopted for this study (i.e., non-tautological framework corresponding to 70% of the answers). Forth, the items were not sent back to the judges. However, the items were thoroughly discussed and approved by the committee of experts as described previously. Another limitation was the fact that the high religious group was composed by followers of a specific religion and the non-religious group was composed by university students. Although this could be a potential bias, this strategy was adopted to guarantee that this sample had high levels of R/S and could be appropriately discriminated from the sample of university students. Likewise, the sociodemographic differences between groups were adjusted in the models as well. It is also important to note that the sample size is slightly smaller than that recommended for carrying out a CFA (Flora and Curran, 2004). For further studies, members and followers of other religions and beliefs, as well as, indigenous, agnostics and atheists should be approached, to verify the instrument’s discriminant capacity. Finally, although several statements emerged from the qualitative analysis, expert meetings have determined the most representative ones and, for this reason, some statements were not included due to repetition, resulting in this brief 11-item instrument.

The Portuguese version of the ARES showed that it is an instrument capable of assessing spirituality through appropriate psychometric properties, which indicates that the ARES can be an important tool for exploring the impact of spirituality on different outcomes and populations. The non-tautological nature of the instrument could serve as a potential reference instrument to be used in research all over the world. Future studies should investigate the feasibility of using this instrument in other cultural backgrounds and other languages.

## Data Availability Statement

The original contributions presented in the study are included in the article/Supplementary Material, further inquiries can be directed to the corresponding author.

## Ethics Statement

The studies involving human participants were reviewed and approved by The Research Ethics Committee of the Medical School of the University of São Paulo. The patients/participants provided their written informed consent to participate in this study.

## Author Contributions

All authors listed have made a substantial, direct and intellectual contribution to the work, and approved it for publication.

## Conflict of Interest

The authors declare that the research was conducted in the absence of any commercial or financial relationships that could be construed as a potential conflict of interest.

## Publisher’s Note

All claims expressed in this article are solely those of the authors and do not necessarily represent those of their affiliated organizations, or those of the publisher, the editors and the reviewers. Any product that may be evaluated in this article, or claim that may be made by its manufacturer, is not guaranteed or endorsed by the publisher.

## Abbreviations

ARES: attitudes related to spirituality scale
BMMRS-P: brief multidimensional measure of religiousness/spirituality
CI: concordance index
CVI: content validity index
FACIT-Sp-12: functional assessment of chronic illness therapy – spiritual well-being scale
ICC: intraclass correlation coefficient
KMO: Kaiser-Meyer-Olkin
SPSS: statistical package for social sciences.
