# Supplementary material for: Development of an Instrument to Assess Spirituality: Reliability and Validation of the Attitudes Related to Spirituality Scale (ARES)
Source: Front Psychol. 2021 Nov 4;12:764132. doi: 10.3389/fpsyg.2021.764132 (PMC8600364; doi:10.3389/fpsyg.2021.764132)
Supplement: Supplementary file 1 [file Table_1.DOCX]

**Additional file 1: Development of the items**

| **Statements** | **Items** |
| --- | --- |
| Relationship with a higher power  Connection with God  Contact with God  Belief in a spiritual reality  Something that teaches us to connect with God  Believing in a higher power  Something linked to faith, not necessarily linked to religion  Something that serves to connect us to God | \| I believe in something sacred or transcendent (God, a higher force). \| \| --- \| |
| Being in touch with God through prayer and other spiritual practices  Have a spiritual experience through practices  Get in touch with God through reading holy books  Individual experience that brings greater awareness of spiritual reality  Inner strength manifested in meditation, contemplation practices  A kind of sensitivity that provides spiritual experiences | \| Meditation, prayer, readings and/or contemplation are practices that I use (at least one of them) to connect with a spiritual force beyond myself. \| \| --- \| |
| It’s seeing something beyond material reality  A force greater than the material world  Belief in something not material  Belief in something greater than material reality  Experiences that manifest a spiritual side | \| I have witnessed facts/situations that have led me to believe that there is something beyond the material world. \| \| --- \| |
| A superior force that helps in everyday life  Something that gives comfort and overcoming on life's difficulties  Higher force that sustains us in difficult times  Higher force to which we cling to explain the facts of life  Faith that sustains in daily life | \| My faith or spiritual beliefs sustain me on a daily basis. \| \| --- \| |
| Connection with other people  Helps me to act towards other people  Something that influences how I relate to other people | \| My spirituality helps me have a better relationship with others. \| \| --- \| |
| Connection between material (physical) and spiritual reality  It influences my life in all aspects, from the physical body to mental health. | \| My spirituality influences my physical and mental health. \| \| --- \| |
| Something that guides us in our actions  Belief in a spiritual world that interferes with people's lives and well-being  A pursuit to be a better person  The experience of spirituality through its actions and behaviors | \| My spirituality encourages me to help others. \| \| --- \| |
| Something that transcends the body  Belief in an afterlife  Belief in the existence of spirit, soul  Belief in reincarnation | \| I believe in continuity after death. \| \| --- \| |
| Doing good to people  Something that makes us better people  Something that teaches us how to deal with the difficulties of everyday life  Believing in the intervention of a higher power in our lives | \| My spiritual beliefs and values guide my day-to-day actions. \| \| --- \| |
| It is related to faith  Something linked to faith, not necessarily linked to religion  Something that gives meaning to existence  Something that gives meaning to daily events | \| My faith or spiritual beliefs give meaning to my life. \| \| --- \| |
| Faith that helps you live better, both physically and spiritually  Personal and spiritual evolution through practices  Doing practices that assist in physical, mental and spiritual development | \| Spiritual practices (e.g., praying, fasting, meditation or other) help maintain or improve my physical or mental health. \| \| --- \| |
