# Supplementary material for: Development of an Instrument to Assess Spirituality: Reliability and Validation of the Attitudes Related to Spirituality Scale (ARES)
Source: Front Psychol. 2021 Nov 4;12:764132. doi: 10.3389/fpsyg.2021.764132 (PMC8600364; doi:10.3389/fpsyg.2021.764132)
Supplement: Supplementary file 2 [file Table_2.DOCX]

**Additional file 2**: **Attitudes Related to Spirituality Scale**


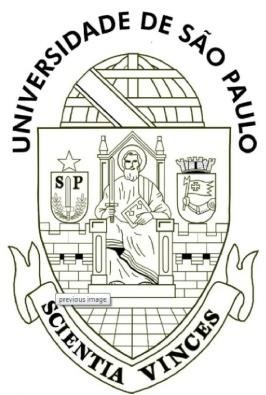


| **ARES**  **Attitudes Related to Spirituality Scale** |
| --- |

Before answering, please read the following instructions:

Spirituality can be understood as a personal search regarding issues related to the sacred, to God and to the meaning of life. Spirituality can manifest itself independently of religion. That is, spiritual people may or may not have religion. Please carefully answer how much you agree or disagree with the statements below on spirituality:

|  | Strongly disagree | Disagree | Neither agree nor disagree | Agree | Strongly agree |
| --- | --- | --- | --- | --- | --- |
| 1. I believe in something sacred or transcendent (God, a higher force). | 1 | 2 | 3 | 4 | 5 |
| 2. Meditation, prayer, readings and/or contemplation are practices that I use (at least one of them) to connect with a spiritual force beyond myself. | 1 | 2 | 3 | 4 | 5 |
| 3. I have witnessed facts/situations that have led me to believe that there is something beyond the material world. | 1 | 2 | 3 | 4 | 5 |
| 4. My faith or spiritual beliefs sustain me on a daily basis. | 1 | 2 | 3 | 4 | 5 |
| 5. My spirituality helps me have a better relationship with others. | 1 | 2 | 3 | 4 | 5 |
| 6. My spirituality influences my physical and mental health. | 1 | 2 | 3 | 4 | 5 |
| 7. My spirituality encourages me to help others. | 1 | 2 | 3 | 4 | 5 |
| 8. I believe in continuity after death. | 1 | 2 | 3 | 4 | 5 |
| 9. My spiritual beliefs and values guide my day-to-day actions. | 1 | 2 | 3 | 4 | 5 |
| 10. My faith or spiritual beliefs give meaning to my life. | 1 | 2 | 3 | 4 | 5 |
| 11. Spiritual practices (e.g., praying, fasting, meditation or other) help maintain or improve my physical or mental health. | 1 | 2 | 3 | 4 | 5 |
