# Supplementary material for: Development of an Instrument to Assess Spirituality: Reliability and Validation of the Attitudes Related to Spirituality Scale (ARES)
Source: Front Psychol. 2021 Nov 4;12:764132. doi: 10.3389/fpsyg.2021.764132 (PMC8600364; doi:10.3389/fpsyg.2021.764132)
Supplement: Supplementary file 3 [file Table_3.DOCX]

**Additional file 3 - Analysis of items of the ARES based on a sample of 170 individuals.**

| **Item** | **Description** | **Item description Cronbach's alpha if the item is deleted** |
| --- | --- | --- |
|  |  |  |
| 1 | I believe in something sacred or transcendent (God, a higher force). | 0.983 |
|  |  |  |
| 2 | . Meditation, prayer, readings and/or contemplation are practices that I use (at least one of them) to connect with a spiritual force beyond myself. | 0.983 |
|  |  |  |
| 3 | I have witnessed facts/situations that have led me to believe that there is something beyond the material world. | 0.984 |
|  |  |  |
| 4 | My faith or spiritual beliefs sustain me on a daily basis. | 0.982 |
|  |  |  |
| 5 | My spirituality helps me have a better relationship with others. | 0.982 |
|  |  |  |
| 6 | My spirituality influences my physical and mental health. | 0.982 |
|  |  |  |
| 7 | My spirituality encourages me to help others. | 0.982 |
|  |  |  |
| 8 | I believe in continuity after death. | 0.983 |
|  |  |  |
| 9 | My spiritual beliefs and values guide my day-to-day actions. | 0.983 |
|  |  |  |
| 10 | My faith or spiritual beliefs give meaning to my life. | 0.982 |
|  |  |  |
| 11 | Spiritual practices (e.g., praying, fasting, meditation or other) help maintain or improve my physical or mental health. | 0.984 |
|  |  |  |
