# Supplementary material for: Development of an Instrument to Assess Spirituality: Reliability and Validation of the Attitudes Related to Spirituality Scale (ARES)
Source: Front Psychol. 2021 Nov 4;12:764132. doi: 10.3389/fpsyg.2021.764132 (PMC8600364; doi:10.3389/fpsyg.2021.764132)
Supplement: Supplementary file 4 [file Table_4.DOCX]

**Additional file 4: Coefficient of agreement frequency for spirituality obtained in the 1st application (test) and 2nd application (retest) of the questionnaire and Kappa values. The columns in the table refer to the test results and the lines, the retest.**

| **Coefficient of agreement** | Strongly  Disagree | Partially disagree | Neither agree nor disagree | Partially  agree | Strongly  agree | Total |
| --- | --- | --- | --- | --- | --- | --- |
|  | **1. I believe in something sacred or transcendent (God, a higher force).** | | | | | |
| Strongly  Disagree | 14 | 1 | 1 | 0 | 0 | 16 |
| Partially disagree | 0 | 4 | 1 | 0 | 0 | 5 |
| Neither agree nor disagree | 0 | 0 | 3 | 1 | 0 | 4 |
| Partially  agree | 1 | 0 | 1 | 8 | 4 | 14 |
| Strongly  agree | 0 | 0 | 0 | 3 | 25 | 28 |
| Total | 15 | 5 | 6 | 12 | 29 | 67 |
| **Kappa (IC 95%) = 0.73 (0.60, 0.87); p-valor<0.001** | | | | | | |
|  | **2. Meditation, prayer, readings and/or contemplation are practices that I use (at least one of them) to connect with a spiritual force beyond myself.** | | | | | |
| Strongly  Disagree | 18 | 2 | 0 | 0 | 0 | 20 |
| Partially disagree | 1 | 5 | 1 | 1 | 0 | 8 |
| Neither agree nor disagree | 0 | 0 | 1 | 2 | 0 | 3 |
| Partially  agree | 0 | 0 | 2 | 10 | 5 | 17 |
| Strongly  agree | 0 | 0 | 1 | 2 | 16 | 19 |
| Total | 19 | 7 | 5 | 15 | 21 | 67 |
| **Kappa (IC 95%) = 0.66 (0.53, 0.80); p-valor<0.001** | | | | | | |
|  | **3. I have witnessed facts/situations that have led me to believe that there is something beyond the material world.** | | | | | |
| Strongly  Disagree | 19 | 2 | 0 | 0 | 0 | 21 |
| Partially disagree | 1 | 4 | 2 | 1 | 0 | 8 |
| Neither agree nor disagree | 0 | 1 | 5 | 1 | 0 | 7 |
| Partially  agree | 0 | 0 | 1 | 11 | 7 | 19 |
| Strongly  agree | 0 | 0 | 0 | 1 | 11 | 12 |
| Total | 20 | 7 | 8 | 14 | 18 | 67 |
| **Kappa (IC 95%) = 0.67 (0.55, 0.80); p-valor< 0.001** | | | | | | |
|  | **4. My faith or spiritual beliefs sustain me on a daily basis.** | | | | | |
| Strongly  Disagree | 21 | 1 | 0 | 1 | 0 | 23 |
| Partially disagree | 0 | 3 | 1 | 1 | 0 | 5 |
| Neither agree nor disagree | 0 | 1 | 3 | 3 | 0 | 7 |
| Partially  agree | 0 | 0 | 2 | 10 | 5 | 17 |
| Strongly  agree | 0 | 0 | 0 | 1 | 14 | 15 |
| Total | 21 | 5 | 6 | 16 | 19 | 67 |
| **Kappa (IC 95%) = 0.68 (0.55, 0.81); p-valor<0.001** | | | | | | |
|  | **5. My spirituality helps me have a better relationship with others**. | | | | | |
| Strongly  Disagree | 20 | 0 | 0 | 1 | 0 | 21 |
| Partially disagree | 2 | 2 | 1 | 1 | 0 | 6 |
| Neither agree nor disagree | 1 | 0 | 6 | 0 | 1 | 8 |
| Partially  agree | 0 | 0 | 2 | 7 | 7 | 16 |
| Strongly  agree | 0 | 0 | 1 | 2 | 13 | 16 |
| Total | 23 | 2 | 10 | 11 | 21 | 67 |
| **Kappa (IC 95%) = 0.63 (0.50, 0.75); p-valor<0.001** | | | | | | |
|  | **6. My spirituality influences my physical and mental health.** | | | | | |
| Strongly  Disagree | 17 | 2 | 0 | 0 | 0 | 19 |
| Partially disagree | 1 | 2 | 0 | 1 | 0 | 4 |
| Neither agree nor disagree | 0 | 2 | 5 | 2 | 4 | 13 |
| Partially  agree | 0 | 0 | 1 | 11 | 7 | 19 |
| Strongly  agree | 0 | 0 | 0 | 2 | 10 | 12 |
| Total | 18 | 6 | 6 | 16 | 21 | 67 |
| **Kappa (IC 95%) = 0.58 (0.45, 0.70); p-valor<0.001** | | | | | | |
|  | **7. My spirituality encourages me to help others.** | | | | | |
| Strongly  Disagree | 19 | 1 | 0 | 0 | 0 | 20 |
| Partially disagree | 1 | 3 | 1 | 1 | 0 | 6 |
| Neither agree nor disagree | 1 | 1 | 2 | 0 | 3 | 7 |
| Partially  agree | 1 | 0 | 2 | 11 | 4 | 18 |
| Strongly  agree | 0 | 0 | 1 | 5 | 10 | 16 |
| Total | 22 | 5 | 6 | 17 | 17 | 67 |
| **Kappa (IC 95%) = 0.57 (0.43, 0.70); p-valor<0.001** | | | | | | |
|  | **8. I believe in continuity after death.** | | | | | |
| Strongly  Disagree | 20 | 1 | 1 | 2 | 0 | 24 |
| Partially disagree | 1 | 0 | 1 | 0 | 0 | 2 |
| Neither agree nor disagree | 3 | 0 | 6 | 3 | 0 | 12 |
| Partially  agree | 0 | 2 | 1 | 8 | 3 | 14 |
| Strongly  agree | 0 | 0 | 0 | 1 | 14 | 15 |
| Total | 24 | 3 | 9 | 14 | 17 | 67 |
| **Kappa (IC 95%) = 0.62(0.49, 0.75); p-valor<0.001** | | | | | | |
|  | **9. My spiritual beliefs and values guide my day-to-day actions.** | | | | | |
| Strongly  Disagree | 19 | 0 | 2 | 2 | 0 | 23 |
| Partially disagree | 2 | 2 | 0 | 0 | 0 | 4 |
| Neither agree nor disagree | 2 | 0 | 5 | 1 | 0 | 8 |
| Partially  agree | 0 | 0 | 7 | 9 | 2 | 18 |
| Strongly  agree | 0 | 0 | 0 | 4 | 10 | 14 |
| Total | 23 | 2 | 14 | 16 | 12 | 67 |
| **Kappa (IC 95%) = 0.56(0.43, 0.70); p-valor<0.001** | | | | | | |
|  | **10. My faith or spiritual beliefs give meaning to my life.** | | | | | |
| Strongly  Disagree | 23 | 2 | 0 | 0 | 0 | 25 |
| Partially disagree | 0 | 2 | 1 | 1 | 0 | 4 |
| Neither agree nor disagree | 3 | 1 | 7 | 3 | 0 | 14 |
| Partially  agree | 0 | 0 | 4 | 6 | 1 | 11 |
| Strongly  agree | 0 | 0 | 1 | 1 | 11 | 13 |
| Total | 26 | 5 | 13 | 11 | 12 | 67 |
| **Kappa (IC 95%) = 0.64(0.51, 0.77); p-valor<0.001** | | | | | | |
|  | **11. Spiritual practices (e.g., praying, fasting, meditation or other) help maintain or improve my physical or mental health.** | | | | | |
| Strongly  Disagree | 16 | 2 | 3 | 1 | 0 | 22 |
| Partially disagree | 1 | 2 | 2 | 1 | 0 | 6 |
| Neither agree nor disagree | 2 | 2 | 3 | 2 | 1 | 10 |
| Partially  agree | 2 | 0 | 3 | 6 | 6 | 17 |
| Strongly  agree | 0 | 0 | 0 | 2 | 10 | 12 |
| Total | 21 | 6 | 11 | 12 | 17 | 67 |
| **Kappa (IC 95%) = 0.42(0.30, 0.55); p-valor<0.001** | | | | | | |
