# Supplementary material for: Development of an Instrument to Assess Spirituality: Reliability and Validation of the Attitudes Related to Spirituality Scale (ARES)
Source: Front Psychol. 2021 Nov 4;12:764132. doi: 10.3389/fpsyg.2021.764132 (PMC8600364; doi:10.3389/fpsyg.2021.764132)
Supplement: Supplementary file 5 [file Table_5.docx]

**Additional file 5: Logistic regression models for the relationship between group (Religious x Medical students) and religious measures adjusted for sociodemographic variables.**

| **Coefficients** | **ARES** | | | **DUREL** | | | **BMMRS** | | |
| --- | --- | --- | --- | --- | --- | --- | --- | --- | --- |
|  | **OR** | **95%CI OR** | **p-value** | **OR** | **95%CI OR** | **p-value** | **OR** | **95%CI OR** | **p-value** |
| Intercept | 0.1 | (0.01, 0.43) | 0.0067 | 0.1 | (0.01, 1.04) | 0.0504 | 0.1 | (0.01, 0.55) | 0.0127 |
| Group (Religious x Medical Students) | 32.1 | (2.94, 515.12) | 0.0076 | 13.7 | (1.14, 294.91) | 0.0561 | 34.2 | (3.18, 582.81) | 0.0069 |
| Age | 1.0 | (0.96, 1.08) | 0.5496 | 1.0 | (0.95, 1.10) | 0.4932 | 1.0 | (0.96, 1.09) | 0.4573 |
| Marital status (married x not married) | 0.6 | (0.12, 2.78) | 0.5475 | 5.5 | (0.92, 46.74) | 0.0788 | 0.5 | (0.10, 2.41) | 0.4385 |
| Education (complete higher education) | 5.4 | (1.48, 20.65) | 0.0112 | 2.0 | (0.40, 8.43) | 0.3408 | 1.6 | (0.36, 6.08) | 0.5069 |
| Income (10 or more salaries) | 1.7 | (0.51, 6.59) | 0.4199 | 0.8 | (0.29, 2.59) | 0.7538 | 0.6 | (0.21, 1.97) | 0.4215 |
| Gender (male) | 0.6 | (0.19, 1.71) | 0.3165 | 0.7 | (0.24, 2.10) | 0.5275 | 1.3 | (0.44, 4.13) | 0.6445 |
